# HOW TO ENGAGE SENIORS AS VOLUNTEERS IN SOCIAL CARE SECTORS: A CASE STUDY OF A TIMEBANK IN HONG KONG

**DOI:** 10.1093/geroni/igac059.3133

**Published:** 2022-12-20

**Authors:** Shiyu Lu

**Affiliations:** City University of Hong Kong, Kowloon, Hong Kong

## Abstract

**Objective:**

Exploring the roles of older adults as volunteers in social care settings has attracted wide attention to facilitate healthy aging. However, knowledge of engaging older adults as volunteers in social care sectors remains scant. This study explores theoretical mechanisms for promoting volunteer engagement among older adults in the social care sector.

**Method:**

This study used a time bank program called Good Hands in Hong Kong as a case study. Good Hands was established in 2018 and engaged almost 200 older adult volunteers to provide buddying and accompany (e.g., home visits and medical escort services) to over 390 frail peers in the community. This study adopted a qualitative method. Three semi-structured focus group interviews with 18 participants, including senior volunteers and the timebank staff, were conducted in January – August 2021. The thematic analysis. Stakeholder checks were conducted in July 2022 to enhance the credibility of the findings.

**Results:**

Three emergent themes were identified as critical components to facilitate engagement among senior volunteers: (1) strong cross-sector collaboration, (2) meaningfulness in voluntary work comprising four subthemes, including capacity optimization, care capacity enhancement, belonging cultivation, and value recognition, and (3) a co-producing environment. In addition, this study also identified the challenges related to the sustainability of the timebank program.

**Conclusion:**

This study is the first to explore mechanisms for promoting volunteer engagement among older adults in the social care sector. The findings provide theoretical and practical implications for the roles of older adults in social welfare production for our society.

